# Comprehensive needs assessment tool for informal cancer caregivers (CNAT-ICs): Instrument development and cross-sectional validation study

**DOI:** 10.1016/j.ijnsa.2024.100240

**Published:** 2024-09-19

**Authors:** Eranthi Weeratunga, Sampatha Goonewardena, Lalitha Meegoda

**Affiliations:** a Department of Nursing, Faculty of Allied Health Sciences, University of Ruhuna, Galle, Sri Lanka; b Department of Community Medicine, Faculty of Medical Sciences, University of Sri Jayewardenepura, Nugegoda, Sri Lanka; c Non-Communicable Disease Research Centre, University of Sri Jayewardenepura, Nugegoda, Sri Lanka; d Department of Nursing and Midwifery, Faculty of Allied Health Sciences, University of Sri Jayewardenepura, Nugegoda, Sri Lanka

**Keywords:** Cancer, CNAT-C scale, Informal caregivers, Needs, Reliability, Sri Lanka, Validity

## Abstract

**Background:**

Growing cancer incidence and its subsequent burden is a worldwide concern. Needs assessment for caregivers has recently received growing attention, as it identifies specific unmet needs. The remaining tools have been established within the healthcare context of Western countries and have been studied only in some Asian populations; it seems appropriate to develop needs assessment tools that apply to a wider ethnic and socio-cultural context.

**Objective:**

This study planned to adapt and examine the psychometric properties of the CNAT-C for the Sri Lankan informal caregivers for wider applicability.

**Design:**

An instrument development and cross-sectional validation study was conducted.

**Setting:**

Apeksha Hospital Maharagama, Sri Lanka (National Cancer Institute).

**Participants:**

A sample of 226 informal caregivers (ICs) providing palliative care for patients with advanced cancer was selected.

**Methods:**

A CNAT-C (41 items; seven factors) was incorporated and used after a cross-cultural adaptation following WHO guidelines after the permission and pilot test. ICs completed the socio-demographic and clinical details along with the validated Centre for Epidemiological Studies-Depression (CES-D), and the World Health Organization-Quality of Life-Brief (WHOQOL-BREF). Internal consistency and test-retest were used to check the reliability. Convergent and divergent validity of the Sinhala version of CNAT (S-CNAT) was confirmed using the CES-D scale and WHOQOL-BREF. Construct validity was evaluated using the exploratory factor analysis (EFA) and confirmatory factor analysis (CFA).

**Results:**

Most of the participants were female (60 %) and married (72 %), and the mean age was 41.78 (SD+14.54). Face and content validity were established during the cross-cultural adaptation. Cronbach's alpha was 0.903 for the overall S-CNAT and the test-retest reliability was 0.965. The S-CNAT was associated positively with the CES-D while negatively with the WHOQOL-BREF. Both EFA and CFA discovered a structure contained seven factors (35 items); domain named as healthcare staff/nurses’ support and information, physical/practical needs, medical officers’ support, psychological needs, social/family support, spiritual/religious support, and hospital facilities/service.

**Conclusions:**

The Sinhala version of CNAT is shown to have adequate validity and reliability in assessing the comprehensive and multidimensional/unmet needs of informal caregivers of patients with advanced cancer (S-CNAT-ICs). It would be a helpful tool to determine the unmet needs of ICs and guide future interventions to meet those needs and enhance or maintain the quality of life for patients and their informal caregivers.

**What is already known**

• Most people with advanced cancer are cared for by their family members or relatives (informal caregivers) in the community; hence, family caregiver competence is of great importance.
• Caregivers’ needs are complicated, and the role of informal caregivers is multi-faceted. The unmet needs of informal caregivers of patients with advanced cancer have recently received growing attention.
• There is no assessment tool focused on the unmet needs of informal cancer caregivers in Sri Lanka.

Alt-text: Unlabelled box

**What this paper adds**

• A new instrument to assess the unmet needs of informal caregivers of patients with advanced cancer has been translated and adopted in Sri Lanka.
• A psychometric analysis demonstrated that the Sinhala version of CNAT-ICs (S-CNAT-ICs) has satisfactory validity and reliability.
• The S-CNAT allows any healthcare professionals especially nurses to assess the unmet needs of patients with any chronic diseases.

Alt-text: Unlabelled box

## 1 Introduction

Cancer is a major condition that affects many people, directly and indirectly, with recurring long-term treatment and a continuing need for care due to the rising prevalence of cancer globally and in Sri Lanka (Bray et al., 2018; ; Cancer Incidence Data- Ministry of Health, 2019; Ministry of Health-Medical Statistical Unit, 2019; GLOBOCAN-World, 2020). Due to the developments in cancer treatment, the illness trajectory and prognosis of cancer have transformed, and patients diagnosed with advanced cancer can live for a prolonged period (Bray et al., 2018; Sung et al., 2020).

Further, essential concerns have been focused on family members/caregivers of patients with cancer according to the WHO's ‘palliative care’ concept in addition to patients (World Health Organization-Palliative care-key facts, 2022). Besides the different impacts on cancer patients, families, and/or their caregivers have faced numerous difficulties when caring for incurable/advanced cancer patients globally irrespective of resources and facilities. Thus, both parties are considered as core parts of care (Shin et al., 2011; Meegoda et al., 2015; Wang et al., 2018; De Silva,2020; Zhu et al., 2021). A caregiver (formal or informal) is anyone who provides care or an individual who helps with physical and psychological care for a person in need (taking care of themselves). Formal caregivers are professional caregivers who are trained and/or paid persons for caregiving activities. However, family caregivers who are not adequately trained or prepared are now providing care for patients could impact high-quality care provision. Yet, the family caregiver is gradually assuming an additional critical role as a vital part of cancer care (National Cancer Institute/NCI, 2020). If the caregiver is a family member (seen among the majority) (Osse et al., 2000) or an unpaid helper who consistently looks after a patient who has chronic illnesses, is elderly, or is a disabled person is called an informal caregiver.

Informal caregivers (ICs) were the individuals responsible for patient care at home conducting specific instructions given by their treating healthcare personnel (Perpiñá-Galvañ, Orts-Beneito, and Fernández-Alcántara, 2019), and engaging many duties of patients such as activities of daily living and instrumental activities of daily living (Adashek and Subbiah, 2020; Zhu et al., 2021). According to De Silva (2020), the name ‘bystander’ was widely used for ICs who look after patients in the hospital as one type of caregiver that was most popular among general people in Sri Lanka is the ‘paid person’. The physical, psychological, and practical effects of a cancer diagnosis are profound for the family caregivers of patients with cancer (Shin et al., 2011) in addition to the other illnesses of caregivers of patients with different chronic illnesses; caregiving of such caregivers badly impacts cardiovascular conditions, cognitive aspects, quality sleep, and psychiatric conditions such as high anxiety and depression among ICs (Vanderwerker et al., 2005; Shin et al., 2011). Further, the quality of life (QoL) of family caregivers was reduced due to financial burdens and workplace issues faced during the illness and its development (Cai et al., 2021; Van Hof et al., 2022). All consequences may lead to negative health outcomes for both cancer patients and their caregivers. Hence, systematic assessment for ICs is crucial to identify the particular problems, needs, resources, and strengths of the caregivers.

Cancer caregivers’ needs are complicated and the role of informal caregivers is multi-faceted (Kim, Carver, and Cannady, 2020; Adejoh, Boele, and Akeju, 2021). According to Osse (2005), a ‘need’ for care has been described as a wish to receive support for an experienced ‘problem’. Another author mentioned that unmet needs could be presented ‘when basic requirements to maintain QoL have not been met and found to be as self-reported form (Ventura, 2004). Many studies have been conducted to identify the unmet needs of ICs of patients with advanced cancer globally and have made numerous efforts to define unmet needs (Ventura, 2004). Needs could be different in various contexts (World Health Organization-Palliative care-key facts, 2022; Wang et al., 2018; Zhu et al., 2021; Adejoh, Boele, and Akeju, 2021; Kim, Carver, and Cannady, 2020; Osse, 2005; Denham et al., 2020). As in the review, seven domains including information, physical, psychological, financial, cancer care service, spiritual, and social needs were identified as unmet needs of ICs (Wang et al., 2018). Similarly, physical, and psychological needs were recognized as the common unmet needs of ICs (Zhu et al., 2021), other concerns related to needs were time, finance, emotions, health, and knowledge of informal caregivers (Zhu et al., 2021; Osse, 2005; Zubaidi et al., 2020).

Although concerned about cancer patients constantly, numerous problems/needs and concerns among ICs should be addressed timely. However, when encountering serious illnesses, meeting needs may become challenging for both patients and caregivers and far more demanding (Ventura, 2004; Glajchen, 2006). Therefore, the identification of needs (unmet needs) of informal caregivers who provide palliative care for patients with advanced cancer would be more important, to plan and continue patient care without facing many challenges. The first step is to identify the problems/needs or concerns of caregivers and provide appropriate care and support, based on such assessment of unmet needs. Further, assessment of needs among ICs is an important step for planning proper support services and attaining caregiver satisfaction. Furthermore, it would detect unmet needs that can be addressed by healthcare professionals and healthcare organizations according to the requirements. To achieve those outcomes, a systematic assessment of the caregiver's needs is crucial and necessary to reduce the gap.

The needs of cancer caregivers were first assessed using the Family Needs Assessment (Tringali, 1986). Then, several scales/tools have been developed and used to assess the unmet needs of ICs worldwide for different patients, caregivers, partners, and survivors such as the 78-item Support Person Unmet Needs Survey (SPUNS) (Campbell, et al., 2009), 40-item Supportive Care Needs Survey for Partners & Caregivers (SCNS-P&C-G) (Sklenarova et al., 2015a), 90-item The Health Care Needs Survey (HCNS) (Hileman, Lackey, and Hassanein, 1992), 90-item Caregivers Needs Scale (CNS) (Longman et al., 1992), 41-item Comprehensive Needs Assessment Tool for Cancer-Caregivers (CNAT-C) (Shin et al., 2011), 72-item Needs Questionnaire (CaTCoN) (Lund et al., 2012), etc. Although we have already initiated the palliative care services in Sri Lanka on a small scale a validated and accurate tool to assess the unmet needs of ICs is not available in Sri Lanka. Therefore, cross-cultural adaptation and assessment of psychometric properties of the previously developed scale would be more easy and important and triggered to fill the current gap due to no validated scales available in Sri Lanka to assess unmet needs/general needs of ICs of patients with advanced cancer which are commonly applicable to the relatively vast majority of cancer types. This study was culturally adapted and examined the psychometric properties of a Sinhala version of the Comprehensive Needs Assessment Tool for Informal Cancer Caregivers (S-CNAT-ICs).

## 2 Methods

### 2.1 Study design and setting

A cross-sectional, descriptive validation study was conducted at the Apeksha Hospital Maharagama which is the main oncology facility situated in Sri Lanka and was acknowledged as the National Cancer Institute in Sri Lanka. Patients all around Sri Lanka are attending the Apeksha Hospital Maharagama for different healthcare requirements.

### 2.2 Study participants

Participants were ICs who are providing palliative care in the oncology wards/units, at the Apeksha Hospital Maharagama for patients diagnosed with advanced cancer (e.g., confirmed stage III, IV, or recurrence cancer-including any type of advanced cancer in the breast, lip/oral, esophagus, thyroid, trachea/lung, bladder, colon, kidney, brain, ovary, pancreas, prostate, rectal, stomach, uterus, etc.). Further, "spouses, blood relatives, and relatives-in-law" who are categorized as family caregivers were considered ICs (Muthucumarana, Samarasinghe, and Elgan, 2018) or spouses/partners, family members, or friends (Stiller et al., 2021). Other inclusion criteria were adult ICs who are 18 years or older with a good understanding of the Sinhala or English language, caring currently for patients with any type of advanced cancer (Cai et al., 2021), and having good physical and mental health. ICs who are providing care for patients with critical conditions due to advanced cancer or other co-morbidities, who have attended training related to caring or were employed (Cai et al., 2021), and who have a history of mental disorders diagnosed by psychiatrists were excluded (Yang et al., 2021).

ICs were selected using a convenient sampling method that met the above inclusion criteria. For that purpose, the admission register of the palliative unit/wards was utilized and the assumption was made that all advanced cancer patients accompanied ICs. The first author checked the eligibility of ICs of such patients who were not involved in the pilot study and all eligible/willing ICs were registered into the register of the principal investigator. The written informed consent from the study participants was obtained after explaining the purpose of the study along with the information sheet. Privacy and confidentiality were maintained throughout the study. After obtaining the written informed consent from ICs, the principal investigator gave questionnaires to consented ICs.

### 2.3 Sample size

The number of items in the scale was considered when calculating the sample size (5–10 subjects per item) (Bland, 2000). There were 41 items on the scale and a 5:1 ratio was taken. The sample size was 205 (41*5). After adding 10 % dropouts, the final sample size was 226 (205+ 20.5).

### 2.4 Study instrument

A pre-tested, interviewer-administered questionnaire was used as the instrument which included socio-demographic (e.g., age, gender, marital status, ethnicity, religion, education level, income level, occupation, caregiving details, sleeping duration, etc.) and clinical characteristics (e.g., self-reported health status, chronic disease conditions, etc.) of the ICs in addition to the details of care recipient/patient. There are different scales/tools to measure different aspects of caregivers as mentioned earlier. However, it was worth measuring several unmet needs using one scale/tool. Thus, the 41-item-Comprehensive Needs Assessment Tool for Cancer-Caregivers (CNAT-C) (Shin et al., 2011) was considered for the current study due to its wide scope, different unmet needs, less comprehensiveness, less time consumed, and easy references as a user-friendly scale than the other scales. If ICs need more time to read/understand and answer questions, it badly affects the patients and reduces the caring time of the patients.

41-item-CNAT-C which consisted of seven domains; health and psychological problems domain (six items), family/social support domain (five items), healthcare staff domain (eight items), informational domain (eight items), hospital facilities/services domain (six items), religious/spiritual support (two items), and practical support domain (six items) (Shin et al., 2011; Mazanec et al., 2018). The health and psychological problems domain assesses needs related to caregiver health, patient concerns, depression, loneliness, anger, and anxiety. The family/social support domain reports the need for help with the overdependence of the patient, absence/lack of gratitude for caregiving, family/interpersonal relationships, and relaxation of caregiver and personal life. Needs associated with connections with physicians, decision-making, communication, and rapport with nurses (e.g., empathy, advice, and consideration for the patient) are measured with the healthcare staff domain. The informational domain estimates need to be associated with information about the patient's illness/tests/treatments, care, hospitals and physicians, financial support, communication, and caregiver stress management as well as complementary and alternative medicine. The domain of needs for religious support is evaluated with two items. The hospital facilities/services domain includes needs for counseling, guidance, space for caregivers, and home care/opportunities to talk with other caregivers. Finally, the practical support domain measures related to transportation, financial assistance, lodging, help with housekeeping and child care, and assistance (Shin et al., 2011; Mazanec et al., 2018).

CNAT-C scale is used in different countries such as South Korea (Shin et al., 2011), China, Singapore (Zhang et al., 2015; Yang et al., 2019), and Australia (Stiller et al., 2021). However, it adapted to the Chinese language (Yang et al., 2019) according to the literature. Satisfactory reliability and validity of CNAT-C were reported in the original study; the Cronbach alpha of the total scale was 0.96 and sub-scales ranged from 0.79 (religious/spiritual support) to 0.95 (health-care staff) (Shin et al., 2011); its seven factors explained 66.4 % of the total variance. Cronbach's alpha for the scale was 0.97 and for sub-scales, it varied from 0.80 to 0.97; 64.2 % of the total variance was explained by seven factors and showed an acceptable level of validity with the 5-level EQ-5D version (EQ-5D-5L/EQ5D) (Shim et al., 2010). Yang et al., (2019) reported high internal consistency for the English and Chinese version scales (more than 0.90) and Cronbach's alpha of seven domains ranging from 0.75 to 0.95; however, showed the low to moderate convergent validity with WHOQOL-BREF. The internal consistency of the Chinese CNAT-C was 0.94 in Zhang et al., (2015); the test-retest reliability was 0.85. Its eight-factor structure was explained as 68.11 % of the total variance.

The scoring format of the original CNAT-C consisted of a Likert/4-point scale (the level to which a need existed within the past month; 0 = no need, 1 = low need, 2 = moderate need, and 3 = high need). A higher score on CNAT-C indicates a higher level of unmet need. CNAT-C domains were calculated by averaging the score for each domain with subsequent linear transformation to a scale of 0–100 based on the EORTC scoring guideline (Shin et al., 2011; Mazanec et al., 2018). CNAT-C was used after obtaining permission from the original authors to change or modify the available items in the scale. Two steps were carried out; cross-cultural adaptation and evaluation of psychometric properties of the CNAT-C according to the standard guidelines as mentioned below.

#### 2.4.1 Step 1- cross-cultural adaptation procedure of CNAT-C

Cross-cultural adaptation guidelines were incorporated in five stages according to the standard guidelines (Beaton et al., 2000; World Health Organization, 2016) forward translation, synthesis, back-translation, expert committee review, and pre-testing. First, an initial translation of the original English version of CNAT-C was done to the Sinhala language by two independent translators who were fluent in both English and their mother tongue (Sinhala language). The above two translators and the principal investigator planned a common meeting to synthesize the results of translations. The consensus report was prepared to incorporate necessary changes. The modified version was then back-translated into the English language by three independent bilingual translators. Face validity was obtained following the above steps. Further, the expert committee consisted of five members in addition to the first author/principal investigator (e.g., oncological experts, palliative care experts, clinicians, professors in community medicine, and senior lecturers in nursing) reviewed both forward and backward translations, and the original version using five consecutive meetings; discussions were continued to reached to group consensus.

Semantic, idiomatic, experiential, and conceptual equivalence were achieved. Semantic equivalence ensures the equivalence of meaning as the translated version needs to mean the same as the original. Idiomatic equivalence warrants the equivalence of idioms that are difficult to translate. Experiential equivalence ensures the experiential quality of the translated questionnaire with regards to the items aiming to capture an experience of daily life that may have differences from the original version. Conceptual equivalence confirms the conceptual meaning replacement that is different from culture to culture (Choi et al., 2012). All the versions of the questionnaire were consolidated and the pre-final version of the questionnaire. However, content validity was ensured throughout the translation and adaptation process using checklists.

All the versions of the questionnaire were consolidated and the pre-final version of the questionnaire. It ensured the content validity of the scale. A pre-final version of the questionnaire was tested using ten ICs, at the palliative care unit as the final step; ICs were selected using selection criteria as mentioned earlier. ICs participated in some health education sessions at the palliative care unit with their patients; however, provided adequate information and obtained written informed consent. The participants were asked to state whether the items were readable and understandable. Some of the wording issues were solved as appropriated into our own culture. The final Sinhala version of the CNAT-C was made for ICs incorporating further face validity (S-CNAT-ICs) and incorporated into the pilot study.

#### 2.4.2 Step II-psychometric properties evaluation

The principal investigator administered all questionnaires for ICs to maintain uniformity of the information. Two psychometric properties were evaluated incorporating reliability and validity. Reliability was assessed using internal consistency and test-retest reliability. The validity of the S-CNAT-ICs was tested using further convergent/divergent validity and construct validity. Face and content validity was obtained when doing the cross-cultural adaptation process. Further, two gold standard scales were incorporated to assess convergent and divergent validity of the S-CNAT-ICs such as the Centre for Epidemiological Studies-Depression scale (20 item-CES-D) (Radloff, 1977) and the World Health Organization-Quality of Life - Brief (26 item-WHOQOL-BREF) (WHO-QOL group, 1988).

The CES-D is a 20-item, short, and self-report scale (Radloff, 1977), which was originally developed to assess depressive symptomatology during the ‘past week’ in the general population worldwide. Each question has four responses from zero (rarely or none of the time) to three (most or all of the time). The total score of the CES-D scale ranges from zero (no depressive symptoms) to 60 (high level of depressive symptoms), where higher scores indicate the presence of more depressive symptomatology. The standard cut-off point that was used to identify those with elevated depressive symptoms was 16 or above on the total scores. This scale was validated and freely used in Sri Lankan studies (Ferdinando, 2006). As reported in the original study, this scale exhibited strong concept validity evidence, good concurrent validity by clinical and self-report criteria, adequate test-retest stability, and high internal consistency (Radloff, 1977). Other studies reported a high Cronbach alpha of 0.855 and the test-retest reliability for the two-week was 0.91 (Chin et al., 2015) for overall CES-D. Also, good reliability was confirmed in another study on suicide attempters and residents; Cronbach's alpha values were 0.940 and 0.895 (Yang, Jia, and Qin, 2015).

The WHOQOL-BREF is a 26-item scale comprised of four domains; physical, psychological, social, and environmental, and originally developed to measure the QoL (WHO-QoL group, 1988). The scale of 24 items, measured four broad domains related to the QoL, namely physical health (seven items), psychological health (six items), social relationships (three items), and environment (eight items); as given in the table above which represented different facets (Table 1) (WHO-QoL group, 1988). Two other items were measured in the global scores; overall QoL (1 item) and overall satisfaction with health (1 item), and a third global QoL score was taken by averaging the above two global items (Agnihotri et al., 2010). The higher values indicated a higher level of QoL. The scale WHOQOL-BREF has been validated in Sri Lankan settings and used freely in many studies (Kumarapeli, Seneviratne, and Wijeyaratne, 2006). As stated in the WHO (WHOQOL User Manual, 1998), Cronbach alpha values for four domains ranged from 0.66 to 0.84; demonstrating good internal consistency and discriminant validity between the ill and well groups. Other studies reported a high Cronbach's alpha for the whole WHOQOL-BREF (0.896) and the domains of the scale were above 0.70; Cronbach's alpha for physical, psychological social relationships, and environmental health were 0.65, 0.77, 0.52 and 0.79, respectively among medical students (Ilic et al., 2019). Cronbach`s alpha among the Norwegian general population was 0.85, 0.83, 0.62, and 0.81, respectively, for the physical, psychological, social, and environmental domains, and 0.92 for the total scale in a study by Kalfoss et al. (2021).

**Table 1:** Socio-demographic profile of ICs (N = 226).

Characteristics/variables	Categories	n	%
Age (years)	18–38	99	43.88
	39–59	98	43.40
	60–80	29	12.80
Gender	Male	91	40.30
	Female	135	59.70
Marital status	Married Unmarried/Single Separated	163 61 02	72.10 27.00 0.90
Education	No schooling	2	0.90
	Grade 1 - Grade 5	16	7.10
	Grade 6–12	188	83.20
	Diploma	9	4.00
	Degree	11	4.90
Family monthly income	1000–5000	56	24.80
(LKR-Sri Lankan Rupees)	5001–10,000	32	14.20
	10,001–49,999	78	34.50
	50,000–99,999	51	22.60
	100,000–200,000	09	4.00
Working status	Currently working	109	48.20
	Currently not working	117	51.80
Occupation (engaged before or currently doing)	Professionals/Executives	20	8.80
	Military personals	06	2.64
	Skilled worker	37	16.28
	Unskilled worker/Laborer	38	16.72
	Self-employed/Business	11	4.84
	Undergraduate/studying	19	8.56
	No occupation	91	40.30
	Retired/pensioner	04	1.79
Total time spent caregiving (months)	Nearly 3	190	84.10
	3–6	14	6.20
	6–12	15	6.60
	12–24	05	2.20
	>25	02	0.80
Weekly time spent on caregiving (hours)	72–90	24	10.60
	91–109	22	9.70
	110–128	60	26.50
	129–147	107	47.30
	148–166	13	5.80
Total time spent sleeping/per day (hours)	1–5	152	67.30
	6–10	74	32.70
Changes of work/job due to caregiving	No change	108	47.80
	Changed job	01	0.40
	Decreased working hours	17	7.50
	Quit/resigned job	12	5.30
	On-leave/temporary leaving	64	28.30
	Retired	01	0.40
	Discontinued/reduced education	23	10.20

Frequency- n; Percentage-%

ICs were informed to complete tools: the S-CNAT-ICs, CES-D, and WHOQOL-BREF which were previously validated in the Sri Lankan context. The construct validity of the S-CNAT-ICs was checked using the exploratory factor analysis (EFA) and confirmatory factor analysis (CFA). The Sinhala version of CNAT-ICs was administered to the sample of 30 ICs in this pilot study at the palliative unit, Teaching Hospital Karapitiya, Galle, Southern Sri Lanka (currently named National Hospital Galle) and these 30 ICs were involved in measuring test-retest reliability two weeks later (Weeratunga, Meegoda, and Goonewardena, 2024).

A subsequent validation study was conducted after the changes of the pilot study incorporating a total of 226 ICs at the oncology wards, Apeksha Hospital Maharagama (Western province Sri Lanka). The principal investigator contacted the sub-set of the same ICs with an interval of two weeks (n = 100 was enrolled to achieve the satisfactory correlation/r of 0.8) (Kennedy, 2022) during the clinic visit.

### 2.5 Method of data collection

The data were gathered after obtaining ethical approval from the Ethics Review Committee, Faculty of Medical Sciences, University of Sri Jayewardenepura (Ref. no. ERC 49/22). Institutional approval (Apeksha Hospital Maharagama and Teaching Hospital Karapitiya) was obtained before the data collection. Consent forms, information sheets, and interviewer-administered questionnaires were given to ICs. The collected data were kept carefully and confidentially. Those who were willing to contribute to the survey voluntarily were employed. Response rate was 100 %.

### 2.6 Statistical analysis

The subject's responses to the item in the questionnaire were scored. All categorical data was coded. Data cleaning and checking were done. Data analysis was done using SPSS version 25.0. The level of significance was accepted at p<0.05. Frequency distribution and basic descriptive statistics were used to describe the result of socio-demographic characteristics and variability of participants.

The reliability of the S-CNAT-ICs was examined using Cronbach's alpha coefficient considering the accepted standard cut-off for internal consistency as 0.70 or above (Nunnally, 1994) considered satisfactory internal consistency. The test-retest reliability was examined using the intra-class correlation coefficient (ICC) using the scores of S-CNAT-ICs in first-time and second-time administrations after two weeks of the first administration. Pearson's correlation coefficient examined the correlations between the domains themselves and the total score of the S-CNAT-ICs to check the construct validity of the S-CNAT-ICs.

Convergent and divergent validity was assessed by item-domain correlation considering the higher correlation of each item with their respective domain and using the Pearson correlation. Correlation coefficient values between 0.10 and 0.29 were considered low, between 0.30 and 0.49 were considered medium, and between 0.50 and 1.00 were considered high and had a very strong correlation (Nunnally, 1994). Both WHOQOL-BREF and CES-D scales were used to examine convergent and divergent validity was assumed that ICs who have met their needs have higher QoL and lower depressive symptoms of ICs.

EFA explores the basic factor structure of S-CNAT-ICs. Factor analysis (FA) was performed using principal component analysis (PCA) with Varimax rotation (Kaiser normalization). The Kaiser-Mayer-Olkin (KMO) and Bartlett's test of Sphericity statistics were examined, and correlation matrix was observed to determine whether FA was average for the data, mostly for sampling adequacy assessment (KMO>0.7), where a value of ≥0.5 of KMO is considered as good sampling adequacy, multi-collinearity assessment (many coefficients in correlation matrix should be 0.3 and above), Bartlett's Test of Sphericity should reach statistical significance (p<0.001) and communalities coefficients should be high (>0.6) (Field, 2013). The number of extracted components was determined by the Scree plot, the percentage of variance explained by each component, the number of Eigenvalues over one (Kaiser-Guttman rule), and consideration of prior psychometric S-CNAT-ICs analysis. Items were considered representative of a component if their item loading was ≥0.40 and in the cross-loading items, the factor, that had a higher loading value, was taken as the respective factor (Field, 2013).

CFA was done using AMOS 23 software (Arbuckle, 2014). The root means square error of approximation (RMSEA) and comparative fit index (CFI) were examined. The cut-off values for acceptable model fit used for this study were: RMSEA˃0.06 for good fit; and CFI˃0.90 for acceptable fit (Arbuckle, 2014). Finally, the factor structure of the S-CNAT-ICs was confirmed.

## 3 Results

### 3.1 Socio-demographic and clinical profile of ICs

In Table 1, the socio-demographics of ICs are exhibited. The mean (±SD) age of ICs was 41.78 (±14.54) and the age range was between 18–76 years. The majority of ICs were females, married, educated up to ordinary and/or advanced level, and earned ≤ Sri Lankan Rupees 50,000/=. Characteristics related to the caregiving of ICs are presented in Table 1.

The majority of ICs had to do caregiving for nearly three months. Nearly 50 % of the sample has spent 129–147 hours per week caregiving 67 % of the sample had a 1–5 hour period for sleep. However, 48 % of ICs had no change in their job due to caregiving tasks. Also, 28 % of participants were on leave of temporary left from their jobs due to caregiving. As a consequence of caregiving, 82 % had not faced any physical illnesses while 58 % of ICs had psychological distress or emotional strain. More than half of the participants had financial strain or pressure due to caregiving. However, the majority confirmed that they had overall good health (82 %). Only 16 % of ICs suffered from chronic medical conditions. Further, they had perceived a high level of social support while perceived family support was at a lower level than expected, and 22 % had no family support.

Most of the patients/care recipients were female and in age 57–77. The three main types/sites of the cancer were breast, colon, and oral, respectively. Nearly, half of the patients with cancer had been diagnosed between 1 to 2 years.

### 3.2 CNAT-C - translations and cross-cultural adaptation

ICs were able to answer the whole tool within 20–30 minutes. Following the consultation after the pilot test, a further revision/modification was incorporated into the scoring system as ‘1 = not required,’ ‘2 = satisfied,’ ‘3 = need is minimal,’ ‘4 = need is moderate,’ and ‘5 = need is high’; some of the scoring patterns (1 = not required, 2 = satisfied) were not in the original scoring system; while filling the interviewer-administered questionnaire, some ICs had not faced such problems, and some needs are not required of them as those needs are already received or satisfied. The final Sinhala version of the S-CNAT was found to be an easily understood, culture-sensitive, and user-friendly tool for informal caregivers (S-CNAT-ICs).

### 3.3 Descriptive statistics and reliability - S-CNAT-ICs, CES-D, and WHOQOL BREF

The mean (SD) of the overall S-CNAT-ICs, test-retest reliability of overall and all domains, and inter-item correlations are included in Table 2. Of the domains: Cronbach's alpha for the Health and Psychological Problems-0.776, Family and social support-0.679, Health Care Staff (Doctors and Nurses)-0.904, Information-0.776, Religious/spiritual support-0.932, Hospital facilities and services-0.689, and Practical needs-0.543.

**Table 2:** Descriptives, reliabilities, inter-item correlations of the overall S-CNAT-ICs and seven domains, and comparison of reliabilities.

Variables	Mean (SD)	
Overall scale (*1st administration*) (N = 226)	114.31 (22.68)	
Domains *(2nd administration)* (n = 100)	Test-retest reliability (by ICC)	Inter-item correlations; *p-value*
Health and Psychological Problems	0.948	0.906; *<0.001*
Family and social support	0.936	0.885; *<0.001*
Health Care Staff (Doctors and Nurses)	0.889	0.814; *<0.001*
Information	0.924	0.875; *<0.001*
Religious/spiritual support	0.899	0.829; *<0.001*
Hospital facilities and services	0.962	0.927; *<0.001*
Practical needs	0.961	0.928; *<0.001*
Total S-CNAT-ICs	0.965	0.945; *<0.001*
**Comparison of reliabilities of S-CNAT-ICs and original scale**		
CNAT	Reliability (Cronbach's alpha)	
	CNAT-C (Shin et al., 2011)	S-CNAT-ICs
Overall scale	0.96	0.903
Domains	Range 0.79 - 0.95	Range 0.54 -0.93
Health and Psychological Problems	0.89	0.776
Family and social support	0.89	0.679
Health Care Staff (Doctors and Nurses)	0.95	0.904
Information	0.90	0.776
Religious/spiritual support	0.79	0.932
Hospital facilities and services	0.85	0.689
Practical needs	0.81	0.543

Intra-class correlation coefficient (ICC); *p* < 0.001. Pearson correlation was used.

**Fig. 1:**
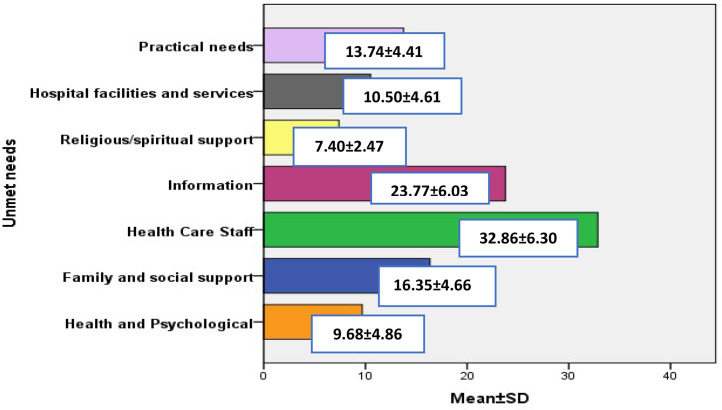
Reported unmet needs among informal cancer caregivers.

Based on the mean scores of seven domains, Health Care Staff (Doctors and Nurses), Information, and Family and social support had the highest mean scores (top three needs) and are included in Fig. 1.

In Table 3, descriptive statistics of the CES-D, WHOQOL-BREF scale, and scores of four domains of the WHOQOL-BREF scale (1st and 2nd administration) are incorporated. Physical QoL scored a higher value than the other three domains.

**Table 3:** Descriptive statistics of the CES-D scale and WHOQOL-BREF scale.

Variables	1st administration				2nd administration			
	Minimum	Maximum	Mean	SD	Minimum	Maximum	Mean	SD
The total score of CES-D	12	55	18.19	8.31	10	47	18.04	7.93
The total score of the WHO-QOL BREF scale	68	105	91.86	7.24	68	133	91.18	7.81
Sub-domains								
Physical QoL	13	35	29.40	3.98	14	35	28.87	3.80
Psychological QoL	15	23	18.73	1.35	15	58	18.77	3.00
Social QoL	4	15	10.53	1.87	3	14	10.42	1.77
Environmental QoL	19	30	24.84	1.98	18	31	24.92	2.16
General Health QoL	2	10	8.36	1.69	2	10	8.19	1.50

Centre for Epidemiological Studies-Depression scale (CES-D), World Health Organization Quality of Life-Brief scale (WHOQOL-BREF)

### 3.4 Reliability of the S-CNAT-ICs and CNAT-C/original scale

Reliabilities of the total S-CNAT-ICs (Cronbach's alpha 0.903) and domains are also included in Table 4. High Cronbach's alpha was reported by the Religious/spiritual needs domain while the Practical needs domain had the lowest Cronbach's alpha, not like in the findings of the original author (Table 2). Therefore, reliabilities were satisfactory for the overall scale (0.90) and other domains except Practical needs. Test-retest reliabilities and inter-item correlation are in Table 2, showing the satisfactory scores for all sub-scales.

**Table 4:** Correlation between CES-D, WHOQOL-BREF, and S-CNAT-ICs.

Scales	Total S-CNAT-ICs		Domains of S-CNAT-ICs						
			Total HPP	Total FSS	Total HCS	Total information	Total RSS	Total HFS	Total PN
C.V.	r	0.486^⁎⁎^	0.498^⁎⁎^	0.221^⁎⁎^	0.147*	0.375^⁎⁎^	0.105	0.434^⁎⁎^	0.482^⁎⁎^
CES-D	*p-value*	*<0.01*	*<0.01*	*<0.01*	*<0.05*	*<0.01*	*0.144*	*<0.01*	*<0.01*
D.V.	r	- 0.646^⁎⁎^	-0.571^⁎⁎^	-0.506^⁎⁎^	-0.272^⁎⁎^	-0.403^⁎⁎^	-0.173^⁎⁎^	-0.415^⁎⁎^	-0.686^⁎⁎^
WHOQOL-BREF	*p-value*	*<0.01*	*<0.01*	*<0.01*	*<0.01*	*<0.01*	*<0.01*	*<0.01*	*<0.01*

CES-D-Centre for Epidemiological Studies-Depression scale; C.V.- Convergent validity; D.V.- Divergent validity; r-correlation coefficient; S-CNAT-ICs-Comprehensive Needs Assessment Tool for Informal Caregivers; WHOQOL-BREF-World Health Organization Quality of Life-Brief scale; Health and Psychological Problems-HPP; Family and social support-FSS; Health Care Staff (Doctors and Nurses)- HCS; Religious/spiritual support-RSS; Hospital facilities and services-HFS; Practical needs-PN; Pearson correlation was used. *p* < 0.05* and *p* < 0.01^⁎⁎^

### 3.5 Validity of the S-CNAT-ICs

CES-D score was positively and significantly associated with total S-CNAT-ICs and scores of domains except Religious/spiritual support (hypothesized that unmet needs are positively correlated with CES-D and negatively correlated with WHOQOL-BREF); depressive symptoms are increased when increasing unmet needs (convergent validity) (Table 4). WHOQOL-BREF score was negatively and significantly associated with total S-CNAT-ICs and scores of all domains; QoL is increased when decreasing needs (divergent validity) (ICs who have met their needs to have higher QoL and lower depressive symptoms of ICs).

The Kaiser–Meyer–Olkin (KMO) measure of sampling adequacy proposes that data seems appropriate for factor analysis (KMO = 0.785). Bartlett's Test of Sphericity reached statistical significance supporting that there is sufficient significant correlation in the data for confirmatory factor analysis (χ^2^ = 7576.346 (595), *p* < 0.001). The majority of the items of commonalities coefficients were high (>0.6).

**Fig. 2:**
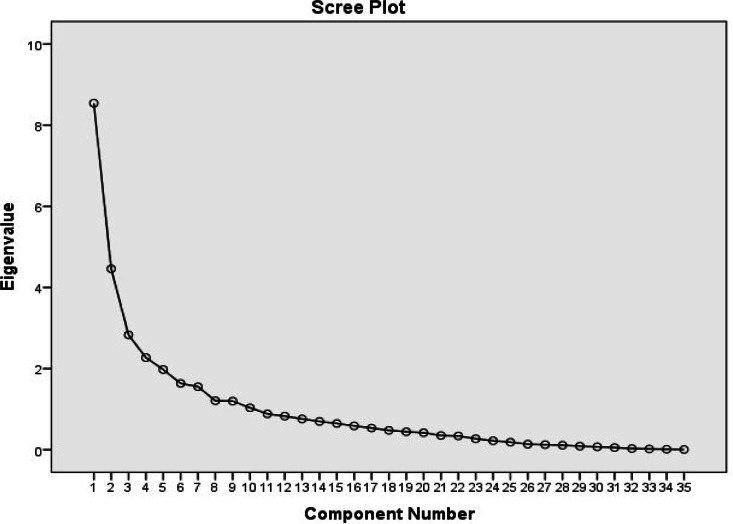
Scree plot – S-CNAT-ICs.

The number of extracted factors/components was confirmed by the Scree plot (Fig. 2), the percentage of variance explained by each component, the number of Eigenvalues over one (Kaiser–Guttman rule), and by considering the original psychometric properties of CNAT-C.

In EFA, some cross-loadings also were reported. EFA extracted a seven-factor model explaining a cumulative variance of 66.48 % (Table 5). Six items were dropped due to their weak primary factor loadings (less than 0.30). Some originally derived items via EFA had to change without considering the loaded factor due to its more relevance to another factor and/or due to several cross-loadings of individual items. No major changes were made to the questionnaire as originally constructed. However, some factors in the original version of the CNAT-C scale were separated and/or altered in the S-CNAT-ICs; health and psychological problems were separated into two factors/domains in the current study and formed physical and psychological domains separately.

**Table 5:** Factor loadings and cross-loadings emerging from EFA of the S-CNAT-ICs.

No.	Factor names of domains and items	Factor loadings						
		1	2	3	4	5	6	7
**1 Healthcare staff/Nurses’ support needs and information needs**								
15	I wished to be actively involved	.839						
16	I wished health care staff to be	.845						
17	I wished sincere interest	.798						
18	I wished my nurses to explain any	.731					*.411*	
19	I wished my nurses to promptly	.727					*.425*	
20	I needed information about	.863						
21	I needed information	.868						
22	I needed information about	.830						
**2 Psychological needs**								
2	I needed help with my concerns		.496					
3	I needed help with depression		.881					
4	I needed help with my feelings		.462					
5	I needed help with loneliness		.903					
6	I needed help with feelings		.855					
27	I needed information about		.465		*.458*			
35	I needed welfare services		.482					
**3 Social and family support needs**								
9	I needed help with difficulties			.843				
10	I needed help with difficulties in interpersonal			.850				
11	I needed help with my own relaxation			.656				
26	I needed help with communication			.655				
36	I needed transportation service			.425				
39	I needed help with my economic burden			.634				
**4 Hospital facilities/service needs**								
23	I needed guidelines or information				.722			
24	I needed information about hospitals				.745			
30	I needed a designated hospital staff member				.648			
32	I needed space reserved for caregivers.				.526			
34	I needed an opportunity to share experiences				.476			
41	I needed assisted care in the hospital or at home.				.432			
**5 Medical officers’ support needs**								
12	I wished to be respected					.959		
13	I wished my doctor to be clear, and specific					.960		
14	I wished to be able to see doctor					.958		
**6 Spiritual/religious support needs**								
28	I needed religious support.						.804	
29	I needed help in finding the						.799	
**7 Physical/practical needs**								
1	I needed help for my own health problems.							.794
33	I needed a visit-home nursing service.				*.473*			.521
37	I needed treatment near my home.							.817
Eigenvalues	8.542	4.459	2.823	2.269	1.975	1.635	1.554	
% Of Variance explained	24.40	12.74	8.09	6.48	5.64	4.67	4.40	
Cumulative %	24.40	37.15	45.24	51.72	57.37	62.04	66.48	
Cronbach's alpha	0.944	0.823	0.778	0.721	0.995	0.932	0.704	

Extraction Method: Principal Component Analysis; Rotation Method: Varimax with Kaiser Normalization**;** Factor loadings: Bold fonts; Cross loadings: Italic fonts.

This physical domain consisted of item 1 and another two items from practical needs (item 37-I needed treatment near my home) and hospital facilities/services (item 33-I needed a visit-home nursing service) domains which were mostly related to the practical needs of ICs. Therefore, named physical/practical needs in the S-CNAT-ICs scale. Previous items of the health and psychological problems (2, 3, 4, 5, and 6) were loaded to the psychological needs. In addition, item 27 “I needed information about caregiving-related stress management” and item 35 “I needed welfare services (e.g., psychological counseling) for caregivers” were classified into psychological factors due to the relevance of other items in the psychological factor of S-CNAT-IC. The healthcare staff factor divided and formed a new factor “medical officers’ support” using three items of healthcare staff-12, 13, and 14.

Other items of healthcare staff and three items of information factors were combined and formed a huge factor named “healthcare staff/nurses’ support needs and information needs” because of its relevance to healthcare professionals/settings gained by ICs. The rest of the items of the information domain moved into hospital facilities/services and social/family support domains. The hospital facilities/services factor was made using several items of some factors such as information, practical needs, and hospital facilities/services domains due to similarities and services obtained/provided from the hospital setting. In the factor of “social and family support”, all relationship matters of ICs, communication, transportation, and relaxation were incorporated. In addition to the already included items (items 9, 10, and 11), one item from Infor (item 26) and two items from Practical needs (items 36 and 39) were loaded. The spiritual/religious domain included the same items of Religious/spiritual support as in the original scale. Overall S-CNAT-ICs and seven domains achieved satisfactory Cronbach's alpha values (Table 5).

**Fig. 3:**
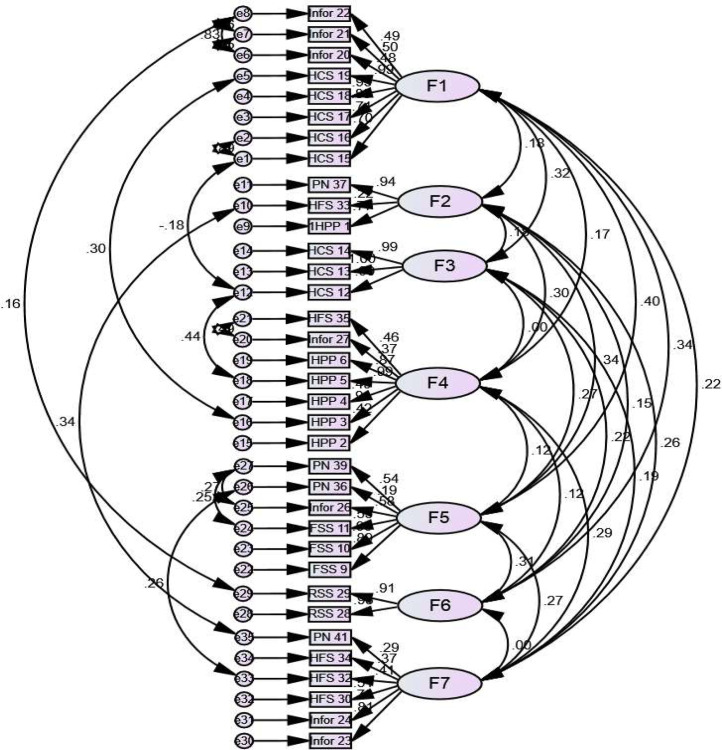
Path diagram of S-CNAT-ICs.

After conducting CFA, the CFI value was 0.936, ≥0.90 demonstrating a good fit of the data. The RMSEA was 0.064. The RMSEA value should be 0.05 ≤ RMSEA ≤ 0.08 to have a good fit. The chi-square divided by the degrees of freedom was 1.908, which should be less than two was the benchmark (the chi-square goodness-of-fit was 1003.598 and the *p*-value was 0.000). Hence, the result indicates that this model is a good fit for the data collected. Finally, a seven-factor structure of the S-CNAT-ICs was confirmed and the path diagram using standardized estimates was presented in Fig. 3.

## 4 Discussion

The current study examined the psychometric properties of the adapted Sinhala version of the CNAT-ICs as the first systematic and comprehensive way to assess the unmet needs of informal cancer caregivers in Sri Lanka (Weeratunga, Goonewardena, and Meegoda, 2024). Other achievements include the adaptation of the CNAT scale for ICs in Sri Lanka (which was developed in another country) due to not having such an assessment currently, and it consisted of numerous dimensions of unmet needs such as physical, psychological, information needs, family/social support, etc. as some of the scales have developed only to measure one or few dimensions (Schur et al., 2015; Sklenarova et al., 2015a). Other strengths include obtaining satisfactory psychometric properties due to the re-production of good reliability and validity of the culturally adapted tool and deriving appropriate factor structure for Sri Lanka using EFA which was confirmed by EFA, scree plot, and CFA. However, CFA was not even conducted in the original study; therefore, limited findings from South Korea (Shin et al., 2011), China (Zhang et al., 2015), and Singapore (Yang et al., 2019) were employed for the discussion of this scale validation. The S-CNAT-ICs were found to be strong. However, a clear and simple tool that can be used to assess the unmet needs of informal cancer caregivers could be filled independently.

### 4.1 Cultural adaptation of S-CNAT-ICs

Adaptation of CNAT-ICs was completed without major issues due to the usage of previously validated tool. Forward-backward translation was done by experts in the oncology field. Cognitive meetings were conducted with the participation of separate palliative and oncology experts in the clinical field in addition to the behavioral scientist. Nursing academics incorporated many opinions/views related to family/informal caregivers’ aspects. During the process, both positive and negative feedback from experts was considered, and a pre-final version was prepared. Due to the participation of multidisciplinary staff and onco-experts, the translation and adaptation procedure was completed smoothly (Choi et al., 2012). A new modification has been incorporated into the scoring system whereas not in the original version. S-CNAT-ICs has a five-point Likert scale (‘1 = not required,’ ‘2 = satisfied,’ ‘3 = need is minimal,’ ‘4 = need is moderate,’ and ‘5 = need is high’), and some of the scoring patterns were not in the original scale (1 = not required, 2 = satisfied) (Shin et al., 2011). Therefore, content and face validity were satisfactorily achieved throughout the procedure.

The original CNAT-C was designed as a self-rating instrument while S-CNAT-ICs in the current study were administered via interviewer (face-to-face); it had both positive (increase understandability) and negative (social desirability bias) effects for ICs. It was difficult to measure the needs of ICs quantitatively using a cross-sectional design. Therefore, a qualitative approach would further benefit the comprehensive assessment of unmet needs in future studies. ICs of patients with different cancer types and ICs who live on island-wide attended for this study increased the generalizability of the study.

### 4.2 Reliability and test-retest reliability of overall S-CNAT-ICs

S-CNAT-ICs showed a good internal consistency for the overall scale (Cronbach's alpha/α 0.903) like the Singapore study (≥0.90 for English and Chinese versions) (Yang et al., 2019) while slightly lower than the Cronbach's alpha of the original scale (0.96) (Shin et al., 2011) and the Chinese study (0.94) (Zhang et al., 2015). The original study (Shin et al., 2011) did not observe the test-retest reliability similar to the Singapore study (Yang et al., 2019). The–test-retest reliability in the current study was at a higher level (ICC-0.935) while a Chinese version had 0.85 test-retest reliability (Zhang et al., 2015). Therefore, the S-CNAT-ICs appear to be a reliable tool to assess the unmet needs of ICs in Sri Lanka as confirmed by previous Cronbach's alpha.

### 4.3 Reliability and test-retest reliability of seven domains

Considering the dimensional Cronbach's α of the S-CNAT-ICs, six domains reported over 0.650 of Cronbach's alpha except for one domain, Practical needs reported 0.543 of Cronbach's alpha (range 0.543–0.932). However, a higher range of dimensional Cronbach's α (range 0.79–0.95) was reported in the original study (Shin et al., 2011) and both versions of the Singapore study (range 0.75–0.95) (Yang et al., 2019). Similar findings were reported in the Chinese study that a high Cronbach's α which ranges from 0.61–0.93 (Zhang et al., 2015). Further, all domains reported a higher range of test-retest (0.742–0.959) in this Sinhalese version which is generally similar to all domains of the Chinese version of CNAT-C (0.80–0.97). Due to the satisfactory reliability and test-retest reliability of domains, could be recommended for future usage.

### 4.4 Convergent and divergent validity of S-CNAT-ICs and its domains

The S-CNAT-ICs displayed good divergent and convergent validity. As anticipated, the correlation between the WHOQOL-BREF values and S-CNAT-ICs domain scores was negative and the association between domain scores of S-CNAT-ICs and the scores of CES-D was positive. Further, the WHOQOL-BREF and domain scores of the S-CNAT-ICs showed significant negative correlations while S-CNAT-ICs and CES-D reported significant positive correlations with all domains except the religious/spiritual support domain. Two studies did not check the convergent validity (Shin et al., 2011; Zhang et al., 2015) while another study checked the convergent validity using the WHOQOL-BREF as the current study (Yang et al., 2019). As reported, there was a weak-moderate association of the CNAT-C scores with WHOQOL-BREF sub-scales, excluding for health and psychological problems domain in the Chinese version and domain 5-religious/spiritual support in both English and Chinese versions (Yang et al., 2019). However, considerably lower correlations were shown in domains 3 (healthcare) and 5 (religious/spiritual) of the current study except for other domains which reported medium to high correlations.

### 4.5 Construct validity of S-CNAT-ICs

Similar to the original study (Shin et al., 2011), EFA revealed a seven-factor structure in the current study that could be another constructive aspect. This structure explained 66.48 % of the total variance and formed 35 items. Although produced a similar number factor structure in both studies, the original CNAT-C scale had 41 items and a rather lower item number derived in the current study. The original author has suggested that future tools with a lower number of items obtained in the current study would be another positive aspect. Factor loadings derived ranged from 0.42 to 0.96 and item communalities values were 0.26–0.98; factor loadings of the original scale ranged from 0.43–0.81 which was mostly similar to the current study while item commonalities were not mentioned in the Shin et al. (2011) study. Another Chinese study (Zhang et al., 2015) formed an eight-factor structure that explains 68.11 % of the total variance which is higher than the current study: 41 items in that study were similar to the original version, factor loadings and item communalities ranged from 0.47–0.85 and 0.54–0.79 with some similarities with the current study and original version. In contrast, factor structure was not developed in a Singapore study (Yang et al., 2019) which limited further comparison.

The S-CNAT-ICs consisted of 35 items which reduced the items to the original scale's items number due to the low factor loadings and some similar items were loaded into one factor, not only that may be due to understanding or meanings or individual perceptions according to the Sri Lankan culture and traditions. As mentioned in the original study, the first CNAT scale had eight domains; physical-psychological domains and family/ social support- religious/ spiritual support were combined into a single domain in contrast to the preparation of the CNAT-C, those domains were separated again into health and psychological problems domains, family/social support and religious/spiritual support domains. However, the current study produced two separate domains for physical and psychological domains may be due to understanding/distinguishing physical vs. psychological problems/issues among ICs than the original study's population.

The original authors mentioned the weakness of their first tool that the inability to differentiate both physical vs. psychological needs whereas the new tool formed two domains for the physical and psychological domains was one strength of the current tool. Another good point was that the physical domain had several items in this version rather than one item of the original scale; it represented some items of practical needs encountered by ICs. Also, some items related to the psychological aspects were loaded to the psychological domain further increasing the support perceived by ICs by the hospital or psychological support services (e.g., stress management program, psychological counseling). Most ICs suffer from financial and emotional strain as reported in the current findings may receive some psychological support from the above-mentioned services as found in previous studies such as negative psychosocial consequences (Sklenarova et al., 2015b; Wang et al, 2018; Zhu et al., 2021).

The previous health care staff domain consisted of items related to doctors and nurses while the current healthcare staff factor consisted of items related to any healthcare personnel and nurses’ support some information needs items were derived into this factor unexpectedly which was not suitable for the health care staff domain and contrast to the two studies (Shin et al., 2011; Zhang et al., 2015). However, the information usually provided by health care staff had some relevance to the health care staff domain. Further, the healthcare staff factor formed a new factor named medical officers’ support, a possible explanation was the medical officers in our system have a hierarchy among all healthcare professionals and mostly adhered to the information given by medical officers (compliance) rather than other professionals, may be reasons for developing a new factor for items 12, 13, and 14. The rest of the items remained and combined some items of information needs. Finally, that large factor was re-named as healthcare staff/Nurses’ support needs and information needs. However, item 25 “I needed information about financial support for medical expenses, either from government and/or private organizations” was deleted due to the lower extraction/coefficient commonalities maybe not receiving such financial support from any type of organization while the two studies had this item in two domains (Shin et al., 2011; Zhang et al., 2015) and other study not studies about the factor structure (Yang et al., 2019).

Similarly, some factor items were separated and/or combined into one factor which differed from the original factors (e.g., social and family support and hospital facilities/services) may be due to cultural differences and understanding/responding variations of ICs in the Sri Lankan context as another study (Zhang et al., 2015). Items 7 and 8 of the previous family and social support domain could not be seen in the new social and family support domain of S-CNAT-ICs. It may be due to not confronting such issues by ICs or could be managed by themselves. Items newly moved into hospital facilities/services (items 23, 24, and 41) also had some connections with this domain while considering different aspects such as further information about medical personnel, treatments, and supportive/assisted care. Although palliative care professionals or onco-experts are providing needy information currently as much as possible information needs to be considered as one of the prior needs in previous studies (Mazanec et al., 2018; Stiller et al., 2021). ICs have to face still some issues related to hospital facilities/services as more prominent issues in developing countries such as Sri Lanka.

Items of religious/spiritual support remained unchanged as in the original scale and reported high Cronbach's alpha as mentioned previously may be due to more exposure to religious activities than previously after the life-threatening diagnosis of their loved ones, also due to the cultural/religious beliefs and connections with their religion which different from other countries. The impact of socio-demographic and clinical characteristics on the unmet needs domain was discussed in the above paragraphs.

### 4.6 Domains of S-CNAT-ICs

Religious/spiritual support of the current study reported the highest Cronbach's α (0.932) and healthcare staff reported Cronbach's alpha of 0.904. Further, religious/spiritual support showed 0.61, 0.79, 0.84, and 0.78 of Cronbach's α in Chinese (Sklenarova et al, 2015a), Korean (Shin et al., 2011), and Singapore (Zhang et al., 2015) studies while the current study obtained the highest Cronbach's α (0.932) than that of others. In contrast to the current study, religious/spiritual support reported the lowest Cronbach's alpha for the Chinese and Korean versions. One possible clarification would be the nature and the religious practices/cultural habits of those natives that they may not had such religious problems/needs or due to lower prevalence (Zhang et al., 2015).

Considering ICs in Sri Lanka, ICs followed more religious/spiritual activities while engaging in caregiving compared to the caregivers in Korea and China where they hoped to get some relief from their respective religions than the previous status. Among participating ICs in the current study, the majority were Buddhist and followed religious rituals (e.g., worship of Lord Buddha, Bhodhi Pooja, etc.) by older ICs than younger usually the trend in Sri Lanka. Due to these reasons and as the coping mechanism (Sklenarova et al., 2015b; Weeratunga et al., 2019), the religious/spiritual domain scored the highest values. The minimal religious relationships and traditions were explained as reasons for the low reliability of the religious/spiritual domain among caregivers of China. As reported in some studies, spirituality is considered to the making sense of one's situation and role rather than a matter of one's relationship with God in Asian cultures (Lee et al., 2015; Hsiao et al, 2022). The patterns and ways of caregiving could be changed according to the cultural differences between Asian and Western societies (developed vs. developing countries) (Leow and Chan, 2017); also due to personal values, attitudes, and fulfillment (Ng et al, 2016).

As mentioned in the original study, the reported highest Cronbach's α was 0.95 for the healthcare staff domain (Shin et al., 2011). In a study by Zhang et al. (2015), the highest Cronbach's alpha was obtained by the availability of healthcare staff (0.93). All domains had more than 0.75 of Cronbach's alpha in both versions of a Singapore study (Yang et al., 2019). Considering four studies, healthcare staff had high Cronbach's alpha for three studies while in the current study, the second highest Cronbach's alpha was reported by healthcare staff; it realizes that unmet needs are usually fulfilled by healthcare staff (e.g., the need for support, information, advice, plans, follow-ups, etc.) in most clinical settings (developed vs. developing) would be more important forever, and caregivers depend on the healthcare team most occasions.

Among most previous caregiver needs assessments, the need for information was one of the major priorities (Campbell et al., 2009) and information items in the current study were classified into the same healthcare staff domain increasing the importance altogether which was proved by other studies (Shin et al., 2011; Zhang et al., 2015; Yang et al., 2019). Male caregivers in the United States of America and cancer caregivers in regional and remote Australia revealed that interaction with the healthcare staff and information needs were some of their main needs (Mazanec et al., 2018; Stiller et al., 2021). When considering participants of the current study, the majority of ICs spent more time on caregiving activities as the main caregiver. This may be the reason for reporting a higher value for healthcare staff as ICs were always linked with the healthcare staff for different purposes/aspects. Literature reported that youngers mostly liked to do jobs, and usual social roles/family responsibilities rather than caregiving as it related to heavy workloads and serious illnesses not experienced during their younger age (Stiller et al., 2021).

The health/psychological domain reported considerably higher values in the current study due to suffering from more psychological distress/emotional strain because of different reasons such as poorer perceived health status/physical ailments, comorbidities, financial issues/low income, etc. similar to the previous study (Shin et al., 2011; Yang et al., 2019). Further, these changes could be triggered due to changes in ICs and/or patients with cancer. Age and gender were influenced by the physical and psychological health of ICs as older persons and females (e.g., maybe with physical comorbidities like diabetes mellites, hypertension, rheumatoid arthritis, weakness/pain, fatigue, lack of sleep, loss of appetite, etc.). Female caregivers were more significantly likely to report overall unmet needs and unmet healthcare needs similar to the current findings (Yang et al., 2019). In contrast, younger caregivers were prone to complain about unmet overall healthcare needs (Stiller et al., 2021) yet very specific unmet needs were more prominent among older caregivers. ICs had to care for older/weak patients with cancer (e.g., having issues related to cancer itself and symptoms, and another disease).

As in the original study, S-CNAT-ICs consisted of seven domains. Most ICs experienced/reported unmet needs were need for healthcare staff, followed by the need for information, family-social support, practical, hospital facilities/services, health-psychological, and religious-spiritual support. However, religious support obtained the highest Cronbach's alpha.

As limitations, future surveys should examine this tool using a larger sample and should study the needs of ICs longitudinally for insights into the diverse needs of ICs as they progress along the cancer journey. Another limitation is the heterogeneity of disease and treatment trajectories, may affect informal caregivers’ unmet needs. Interviewer-administered questionnaires and face-to-face discussions may cause social desirability bias, mainly when talking about sensitive topics or mental health issues; interviewer-administered questionnaires impacted participants in different ways and need to be selected for better ways of administration (self or interviewer) in future studies to minimize those biases. Also, using an instrument that is not sensitive to the types of informal caregivers may lead to unnecessary participant burden and unable to obtain needy information. If this tool starts further testing in the future about domains comprised of lower findings, would be important for informal cancer caregivers.

## 5 Conclusions

The purpose of this study was to adapt and examine the psychometric properties of the Sinhala version of CNAT for informal cancer caregivers (S-CNAT-ICs) in Sri Lanka. To the best of the authors’ knowledge, the current study is the first systematic and comprehensive effort to estimate the needs of ICs of Sri Lankan patients with cancer which is a multidimensional tool.

The authors endorse that the most possibly beneficial tool for both research and clinical use future. The S-CNAT-ICs constitute a meaningful and valid response to the challenges of cancer care, permitting the assessment of needs in cancer caregivers with a comprehensive yet brief and psychometrically sound tool. Also, it would apply to the vast majority of caregivers and would support to preparation of interventions for ICs based on their needs and enable further research. Due to the burden of patients with cancer, the feasibility of a ‘user-friendly’ tool in terms of length and time is also important in the field of oncology while addressing comprehensive needs among ICs. Additionally, its self-report plan enables straight assessment of caregivers’ needs and decreases administrative burden. Application of the S-CNAT-ICs might be feasible in our countries with a socio-cultural context and healthcare system similar to the status of Asian countries. Despite limitations, results suggest that the CNAT constitutes a meaningful and valid response to the challenges faced in cancer care, making the best of existing tools and identifying needs. Therefore, priority setting in national cancer care policy or resource allocation is done in an evidence-based and patient-centered manner.

## Ethical approval

The Ethics Review Committee, Faculty of Medical Sciences, University of Sri Jayewardenepura, Sri Lanka approved the study (reference numbers: ERC 49/22). All participants provided written informed consent.

## Availability of data and materials

All individual data is labeled using codes, the data set is protected using a password, and electronic data will be stored for 10 years. The questionnaires on paper will be stored for five years. All data are available from the corresponding author upon reasonable request and some data are available as supplementary files.

## Funding sources

This research did not receive any specific grant from funding agencies in the public, commercial, or not-for-profit sectors.

## CRediT authorship contribution statement

**Eranthi Weeratunga:** Writing – original draft, Visualization, Validation, Resources, Methodology, Investigation, Formal analysis, Data curation, Conceptualization. **Sampatha Goonewardena:** Writing – review & editing, Validation, Supervision, Methodology, Conceptualization. **Lalitha Meegoda:** Writing – review & editing, Validation, Supervision, Methodology, Conceptualization.

## Declaration of competing interest

The authors declare that they have no known competing financial interests or personal relationships that could have appeared to influence the work reported in this paper.

The author is an Editorial Board Member/Editor-in-Chief/Associate Editor/Guest Editor for *[Journal name]* and was not involved in the editorial review or the decision to publish this article.

## Appendix Supplementary materials

Image, application 1

Image, application 2
