# Supplementary material for: Comprehensive needs assessment tool for informal cancer caregivers (CNAT-ICs): Instrument development and cross-sectional validation study
Source: Int J Nurs Stud Adv. 2024 Sep 19;7:100240. doi: 10.1016/j.ijnsa.2024.100240 (PMC11465200; doi:10.1016/j.ijnsa.2024.100240)
Supplement: Supplementary file 1 [file mmc1.pdf]

පිළිකා රෝගීන් රැකබලා ගන්නන්/අවිධිමත් සත්කාරකයන් සඳහා වූ පරිපූර්ණ අවශ්‍යතා ඇගයුම් මෙවලම (35-item Sinhalese Version of Comprehensive Needs Assessment Tool for informal cancer caregivers/CNAT- ICs)

අතිශයින් බරපතල/දිගු කාලීන පිළිකා ඇති රෝගීන් රැකබලා ගන්නන්ගේ/අවිධිමත් සත්කාරකයන්ගේ (informal cancer caregivers) අවශ්‍යතා තක්සේරු කිරීමේ මෙවලමකි. කරුණාකර සෑම ප්‍රකාශයක්ම හොඳින් කියවා, පසුගිය මාසය තුළ ඔබගේ රෝගියාගේ පිළිකාව හේතුවෙන් ඔබට ඕනෑම ස්ථානයකදී හෝ අවස්ථාවකදී මුහුණ දීමට සිදුවූ, ඔබේ අත්දැකීම වලට අනුව පිළිතුරු දෙන්න.

මෙය කාණ්ඩ 7 කින් සහ අයිතම 35 කින් යුක්ත වේ: සෞඛ්‍ය කාර්ය මණ්ඩලය/හෙදියන්ගේ සහාය අවශ්‍යතා සහ තොරතුරු අවශ්‍යතා (අයිතම 08), ශාරීරික/ප්‍රායෝගික අවශ්‍යතා (අයිතම 03), වෛද්‍ය නිලධාරීන්ගේ සහාය අවශ්‍යතා (අයිතම 03), මනෝවිද්‍යාත්මක අවශ්‍යතා (අයිතම 07), පවුල සහ සමාජ සහාය අවශ්‍යතා (අයිතම 06), අධ්‍යාත්මික/ආගමික සහාය අවශ්‍යතා (අයිතම 02), සහ රෝහල් පහසුකම්/සේවා අවශ්‍යතා (අයිතම 06).

සෑම කරුණක් සඳහාම, ප්‍රකාශය ඔබට අදාල නොවේ නම් හෝ ඔබට එවැනි අපහසුතාවයක් නොමැති නම් හෝ ඔබට එවැනි අපහසුතාවයක් ඇතත් ඔබගේ රෝගියා දැනටමත් උපකාර ලබමින් සිටින බැවින් පහසුකමෙහි අවශ්‍යතාවයක් නැති බැවින් **"අවශ්‍ය නොවුණි"** හෝ **"සෑහීමට පත්"** යන්න හරි ලකුණින් (✓) සලකුණු කරන්න **(1=අවශ්‍ය නොවුණි, 2=සෑහීමට පත්)**.

නමුත්, ඔබට අදාල කරුණ සම්බන්ධයෙන් පහසුකමක් අවශ්‍ය නම් **"අවශ්‍යයතාවය අවමයි"**, **"අවශ්‍යයතාවය මධ්‍යමයි"**, **"අවශ්‍යයතාවය ඉහළයි"** යන මට්ටම් තුන අතරින් අවශ්‍යතාවය කෙතරම් ද යන්න හරි ලකුණින් (✓) සලකුණු කර ඔබගේ අවශ්‍යතා මට්ටම වඩාත් හොඳින් පිළිබිඹු කරන අංකය තෝරන්න (**3 =අවශ්‍යයතාවය අවමයි, 4=අවශ්‍යයතාවය මධ්‍යමයි, 5=අවශ්‍යයතාවය ඉහළයි**). සංඛ්‍යාව වැඩි වන තරමට ඔබේ අවශ්‍යතා මට්ටම ඉහළ යයි.

උදාහරණයක් ලෙස, පහත ප්‍රකාශය සඳහා ('ඔබගේ රෝගියාට නැවත පිළිකාව ඇතිවීමේ බිය සමඟ සාර්ථකව කටයුතු කිරීමට මට උදව් අවශ්‍යය'). මෙය ඔබට අදාළ නොවන බව ඔබ සිතන්නේ නම් හෝ ඔබට මේ සම්බන්ධයෙන් කිසිදු ගැටළුවක් නැති නම් හෝ ඔබට ගැටළුවක් ඇතත් මේ සම්බන්ධයෙන් උදව් ලබාගෙන ඇති බැවින් උදව් ලබාගැනීමේ අවශ්‍යතාවයක් දැනට නැතිනම්, පහත පරිදි 'අවශ්‍ය නොවේ' යන්න හරි ලකුණින් (✓) සලකුණු කරන්න.

පසුගිය මාසය තුළ...	අවශ්‍ය නොවුණි 1	සෑහීමට පත් 2	අවශ්‍යයතාවය		
			අවමයි 3	මධ්‍යමයි 4	ඉහළයි 5
14. ඔබගේ රෝගියාට නැවත පිළිකාව ඇතිවීමේ බිය සමඟ සාර්ථකව කටයුතු කිරීමට මට උදව් අවශ්‍ය විය.	✓				

ඔබගේ රෝගියාට නැවත පිළිකාව ඇතිවීමේ බිය සමඟ කටයුතු කිරීමේ දුෂ්කරතා අත්විඳ ඇත්නම් සහ ඒ සම්බන්ධයෙන් උදව් ලබාගැනීමේ අවශ්‍යතාවය ඉතා වැඩි ලෙස දැනුනි නම් පහත පරිදි අංක 5 කොටුව (✓) සලකුණු යොදන්න.

පසුගිය මාසය තුළ...	අවශ්‍ය නොවුණි 1	සෑහීමට පත් 2	අවශ්‍යයතාවය		
			අවමයි 3	මධ්‍යමයි 4	ඉහළයි 5
14. ඔබගේ රෝගියාට නැවත පිළිකාව ඇතිවීමේ බිය සමඟ සාර්ථකව කටයුතු කිරීමට මට උදව් අවශ්‍ය විය.					✓

පසුගිය මාසය තුළ.......		අවශ්‍ය නොවුණි	සෑහීමට පත්	අවශ්‍යතාවය		
				අවමයි	මධ්‍යමයි	ඉහළයි
		1	2	3	4	5
**සෞඛ්‍ය කාර්ය මණ්ඩලය/හෙදියන්ගේ සහාය අවශ්‍යතා සහ තොරතුරු අවශ්‍යතා (අයිතම 08)**						
1	රෝගියා සම්බන්ධ වෛද්‍ය පරීක්ෂණ හෝ ප්‍රතිකාර සම්බන්ධයෙන් තීරණ ගැනීමේ ක්‍රියාවලියට මා සක්‍රීය ලෙස සහභාගී කරගනු දැකීම මට අවශ්‍ය විය.					
2	සෞඛ්‍ය අංශයේ පිරිස් ඔවුනොවුන් අතර මනා සම්බන්ධීකරණයක් සහ සන්නිවේදනයක් පවත්වා ගනු දැකීම මට අවශ්‍ය විය (වෛද්‍යවරුන් සහ/හෝ වෛද්‍යවරුන් සහ හෙදියන් අතර).					
3	හෙදියගෙන් අවංක උනන්දුවක්, සුහද බවක් සහ සහකම්පනයක් (empathy) මා කෙරෙහි පවතිනු දැකීම මට අවශ්‍ය විය.					
4	හෙදියන් රෝගියාට ලබාදෙන කිසියම් ප්‍රතිකාරයක හෝ සත්කාරයක අරමුණු පැහැදිලි කරදෙනු දැකීම මට අවශ්‍ය විය.					
5	හෙදියන් රෝගියාගේ අපහසුතාවයන්ට සහ වේදනාවන්ට වඩා ඉක්මනින් ප්‍රතිචාර දක්වනු ලැබීම මට අවශ්‍ය විය.					
6	රෝගියාගේ රෝගයේ වත්මන් තත්ත්වය සහ ඉදිරි කාලය තුලදී විය හැකි දේ සම්බන්ධයෙන් මට තොරතුරු අවශ්‍ය විය.					
7	රෝගියාගේ වෛද්‍ය පරීක්ෂණ, ප්‍රතිකාර, සහ ඒවායේ අතුරු අබාධ සම්බන්ධයෙන් මට තොරතුරු දැන ගැනීම අවශ්‍ය විය.					
8	නිවසේදී රෝගියාට සත්කාර කළ යුතු ආකාරය (නිද: රෝග ලක්ෂණ, ආහාර, ව්‍යායාම ආදිය) සම්බන්ධයෙන් මට තොරතුරු අවශ්‍ය විය.					
**ශාරීරික/ප්‍රායෝගික අවශ්‍යතා (අයිතම 03)**						
9	මගේ ශාරීරික රෝගී තත්ත්ව සම්බන්ධයෙන් මට උපකාර අවශ්‍ය විය.					
10	මගේ රෝගයකදී මගේ නිවස අවටින් ම මට ප්‍රතිකාර ලබා ගැනීමට අවශ්‍ය විය.					
11	රෝගියා නිවසේ සිටින අවස්ථාවකදී නිවසට පැමිණ සාත්තු කරන හෙද සේවයක් මට අවශ්‍ය විය.					
**වෛද්‍ය නිලධාරීන්ගේ සහාය අවශ්‍යතා (අයිතම 03)**						
12	වෛද්‍ය කාර්යය මණ්ඩලය විසින් මා පුද්ගලයකු ලෙස සලකනු ලැබීම සහ ගරුත්වයෙන් යුතුව කටයුතු කරනු දැකීම මට අවශ්‍ය විය.					

පසුගිය මාසය තුළ.......		අවශ්‍යා නොවුණි	සෑහීමට පත්	අවශ්‍යයතාවය		
				අවමයි	මධ්‍යමයි	ඉහළයි
		1	2	3	4	5
13	වෛද්‍ය කාර්යය මණ්ඩලය රෝගියා සම්බන්ධ විස්තර ලබා දීමේදී පැහැදිලිව, නිශ්චිතව සහ අවංකව සපයනු දැකීම මට අවශ්‍ය විය.					
14	මා හට අවශ්‍ය වූ විට වෛද්‍යවරයා ඉක්මනින් සහ පහසුවෙන් මුණගැසීමට අවශ්‍ය විය.					
	**මනෝවිද්‍යාත්මක අවශ්‍යතා (අයිතම 07)**					
15	රෝගියාගේ කටයුතු කිරීමේදී මගේ මානසික අවපීඩනය/ව්‍යාකූලත්වය (depression) සම්බන්ධයෙන් මට උපකාර අවශ්‍ය විය.					
16	රෝගියාගේ කටයුතු කිරීමේදී මගේ තරහව (anger), නොරුස්සන බව සහ නොසන්සුන්බව සම්බන්ධයෙන් මට උපකාර අවශ්‍ය විය.					
17	රෝගියා සමග සිටීමේදී මගේ තනිකම හෝ හුදෙකලා හැඟීම් (loneliness) සම්බන්ධයෙන් මට උපකාර අවශ්‍ය විය.					
18	රෝගියාගේ කටයුතු කිරීමේදී නිශ්චිතව කිව නොහැකි ආකාරයේ මගේ කාංසා හැඟීම් (anxiety) සම්බන්ධයෙන් මට උපකාර අවශ්‍ය විය.					
19	රෝගියාගේ කටයුතු කිරීමේදී මගේ සිතේ ඇති වෙන රැක බලා ගැනීම සම්බන්ධ ආතති කළමනාකරණය (stress) පිළිබඳව මට තොරතුරු අවශ්‍ය විය.					
20	රැකබලා ගන්නන් සඳහා වූ සුභසාධන සේවා සඳහා මඟ පෙන්වීමක් හෝ උපදෙස් ලබා දීමක් (නිද: මනෝවිද්‍යාත්මක උපදේශනය) මට අවශ්‍ය විය.					
21	මම රෝගියා සම්බන්ධව සැලකිලිමත් වන ආකාරය පිළිබඳව මට උපකාර අවශ්‍ය විය.					
	**පවුල සහ සමාජ සහාය අවශ්‍යතා (අයිතම 06)**					
22	පිළිකාව හඳුනාගැනීමෙන් අනතුරුව රෝගියා සමග අන්තර් පුද්ගල සබඳතා පවත්වා ගැනීමේ ගැටලු සම්බන්ධයෙන් මට උපකාර අවශ්‍ය විය.					
23	පිළිකාව හඳුනාගැනීමෙන් අනතුරුව රෝගියාගේ පවුල තුල සබඳතා පවත්වාගැනීමේ ගැටලු සම්බන්ධයෙන් මට උපකාර අවශ්‍ය විය.					
24	රෝගියා සහ/හෝ පවුලේ වෙනත් සාමාජිකයන් සමග සුහදව තොරතුරු හුවමාරු කර ගැනීම සම්බන්ධයෙන් මට උපකාර අවශ්‍ය විය.					
25	මගේ විවේකය සහ පෞද්ගලික කටයුතු/ජීවිතය පවත්වා ගැනීම (work and home care) සම්බන්ධයෙන් මට උපකාර අවශ්‍ය විය.					

	පසුගිය මාසය තුළ.......	අවශ්‍ය නොවුණි	අවශ්‍යයතාවය			
			සෑහීමට පත්	අවමයි	මධ්‍යමයි	ඉහළයි
		1	2	3	4	5
26	රෝගියා මගේ පවුලේම අයෙකු නම්, පිළිකාව හේතුවෙන් මුහුණදීමට සිදු වූ ආර්ථික අපහසුතා (නිද: ප්‍රතිකාර සඳහා වියදම්, ආදායම් අහිමි වීම) දරාගැනීමට මට උපකාර අවශ්‍ය විය.					
27	මට රෝහලට ඒමට සහ නිවසට යාමට ප්‍රවාහන සේවාවක් අවශ්‍ය විය.					
අධ්‍යාත්මික/ආගමික සහාය අවශ්‍යතා (අයිතම 02)						
28	මා අනුගමනය කරනු ලබන/අදහන ආගම අනුව ආගමික සහයෝගය (නිද: ආගම මෙනෙහි කිරීම, ආගමානුකූලව සිතීම) මට අවශ්‍ය විය.					
29	රෝගියාට රෝගියාගේ ජීවිතය/ වර්තමාන තත්වය සම්බන්ධයෙන් තේරුම සොයා දීමට මට උපකාර අවශ්‍ය විය.					
රෝහල් පහසුකම්/සේවා අවශ්‍යතා (අයිතම 06)						
30	පිළිකාවට ප්‍රතිකාර ලබාගත හැකි සායන සහ ප්‍රතිකාර කරන වෛද්‍යවරුන් සම්බන්ධයෙන් මට තොරතුරු අවශ්‍ය විය.					
31	රෝගියාට ලබා දීමට සුදුසු වෙනත් වෛද්‍ය ක්‍රම සහ ප්‍රතිකාර සම්බන්ධයෙන් මට මාර්ගෝපදේශ හෝ තොරතුරු අවශ්‍ය විය.					
32	රෝගියා රැකබලා ගැනීමේ කාලයේදී කවර හෝ ගැටළුවක් නිරාකරණය කර ගැනීම සඳහා නිශ්චිතව නම් කරන ලද රෝහල් කාර්ය මණ්ඩලයේ සාමාජිකයකුගේ උපකාර මට අවශ්‍ය විය.					
33	රෝහලේ දී හෝ නිවසේදී හෝ රෝගියා සම්බන්ධයෙන් සත්කාර කිරීමේ දී මට සහාය/සහායකයන් අවශ්‍ය විය.					
34	වෙනත් රෝගීන් රැකබලා ගන්නන් සමග අත්දැකීම් සහ තොරතුරු බෙදාගැනීමට අවස්ථාවක් මට අවශ්‍ය විය.					
35	රැකබලා ගන්නන් සඳහාම වෙන්කළ ස්ථානයක් (නිද: ආහාර ගැනීමට සහ විවේක ගැනීම) මට අවශ්‍ය විය.					

ඔබගේ සහභාගීත්වයට ස්තුතියි!!!
