# Supplementary material for: Comprehensive needs assessment tool for informal cancer caregivers (CNAT-ICs): Instrument development and cross-sectional validation study
Source: Int J Nurs Stud Adv. 2024 Sep 19;7:100240. doi: 10.1016/j.ijnsa.2024.100240 (PMC11465200; doi:10.1016/j.ijnsa.2024.100240)
Supplement: Supplementary file 2 [file mmc2.pdf]

박종혁

From:jonghyock@gmail.com
To:eranthiweeratunga@yahoo.com
Wed, Apr 5, 2023 at 4:16 AM

OK thank you
Park

박종혁 드림

* 음성 받아쓰기를 사용해 오탈자/띄어쓰기 오류가 있을 수 있어 양해 부탁드립니다.

2023. 4. 5. 오전 1:46, eranthiweeratunga@yahoo.com 작성:

Dear Sir/Madam,
I hope you all are doing well.
I am Eranthi Weeratunga, currently working as a senior lecturer in the Department of Nursing, Faculty of Allied Health Sciences, University of Ruhuna, Sri Lanka.
I have started my Ph.D. in the palliative care field under the supervision of Dr. (Mrs.) Lalitha Meegoda, Department of Nursing and Midwifery, Faculty of Allied Health Sciences, University of Sri Jayewardenepua, and Prof. Sampatha Goonawardane, Consultant Community Physician/Profession in Community Medicine, Department of Community Medicine, Faculty of Medical Sciences, University of Sri Jayewardeneura, Sri Lanka.
As one of the objective of the study, I have to assess the unmet needs of informal caregivers of patients with Advanced cancer in Sri Lanka. I was able to find the below manuscript.

The development of a comprehensive needs assessment tool for cancer-caregivers in patient-caregiver dyads

Shin DW, Park J-H, Shim E-J, Park J-H, Choi J-Y, Kim S G, Park E-C. (2010).

After your permission, I have included the CNAT-C scale into my PhD project.
Today, Modified Delphi study was conducted incorportaing resource persons from the cancer and pallitive care settings in Sri Lanka. However, we were able to find some concerens related to several items that they were not very familer to the Sri Lankan setting and current prectices, may be due to the changes of culture, traditions in Sri Lanka, etc.
To solve that issue, we may need to add some words or explantion to original items of the CNAT-C scale.
I would like to request your permission to modifiy such items in the original scale.
If possible, I would like to have a zoom discussion with your memebrs and my resource persons.
I am looking forward to your kind response.
Thank you
Best Regards
Eranthi

Ms. SMEB Weeratunga
RN, BScN, MPhil
Senior Lecturer
Department of Nursing
Faculty of Allied Health Sciences
University of Ruhuna
Mobile No. - +94 77 2258519, +94 71 4402662
E-mail-
eranthiweeratunga@yahoo.com
eranthiw@ahs.ruh.ac.lk

eranthiw@ahs.ruh.ac.lk
From:eranthiw@ahs.ruh.ac.lk
To: Eranthi Weeratunga
Fri, Jul 1, 2022 at 11:28 AM

Thank you
Best Regards
Eranthi

Ms. SMEB Weeratunga
RN, BScN, MPhil
Senior Lecturer
Department of Nursing
Faculty of Allied Health Sciences
University of Ruhuna

Mobile No. -
+94 77 2258519, +94 71 4402662
E-mail- eranthiweeratunga@yahoo.com, eranthiw@ahs.ruh.ac.lk

----- Forwarded Message -----
From: Kyoung Eun Yeob <gy10060624@gmail.com>
To: "eranthiw@ahs.ruh.ac.lk" <eranthiw@ahs.ruh.ac.lk>
Cc: Jong Hyock ParK <jonghyock@gmail.com>
Sent: Friday, July 1, 2022 at 10:32:07 AM GMT+5:30
Subject: Fwd: Permission to validate use in Sri Lanka

I have emailed you the assessment scale you requested, so please check it.

Best Regards

Kyoung Eun Yeob, Ph.D Major in Health Policy
Associate Professor
C.B.N.U Institutes of Health & Science Convergence
Chungbuk Public Health Policy Institute
Chungbuk Tobacco Control Center
Address: 1, Chungdae-ro, Seowon-gu, Cheongju-si 28644 Korea
Office: +82-43-262-9030, **Phone:** +82-10-4877-1059
Homepage: http://www.cbnuihsc.kr/

---------- Forwarded message ---------
보낸사람: **박종혁** <jonghyock@gmail.com>
Date: 2022년 6월 28일 (화) 오후 7:56

Subject: Fwd: Permission to validate use in Sri Lanka
To: 엽경은 <gy10060624@gmail.com>
박종혁 드림
* 음성 받아쓰기를 사용해 오탈자/띄어쓰기 오류가 있을 수 있어 양해 부탁드립니다.
전달된 메시지 시작:

보낸 사람: eranthiw@ahs.ruh.ac.lk
날짜: 2022년 6월 28일 오전 1시 9분 4초 GMT+9
받는 사람: Jonghyock@gmail.com, whitemiso@ncc.re.kr
제목: Permission to validate use in Sri Lanka
답장받는 사람: "eranthiw@ahs.ruh.ac.lk" <eranthiw@ahs.ruh.ac.lk>

Dear Sir/Madam,
I am Eranthi Weeratunga, currently working as a senior lecturer in the Department of Nursing, Faculty of Allied Health Sciences, University of Ruhuna, Sri Lanka.

I hope to start my Ph.D. in the palliative care field under the supervision of Dr. (Mrs.) Lalitha Meegoda, Department of Nursing and Midwifery, Faculty of Allied Health Sciences, University of Sri Jayewardenepua, and Prof. Sampatha Goonawardane, Consultant Community Physician/Profession in Community Medicine, Department of Community Medicine, Faculty of Medical Sciences, University of Sri Jayewardeneura, Sri Lanka.
For that purpose, I hope to assess the unmet needs of informal caregivers of patients with Advanced cancer in Sri Lanka.
I was able to find the below manuscript and hope your assessment scale may suit my study.
The development of a comprehensive needs assessment tool for cancer-caregivers in patient-caregiver dyads
Shin DW, Park J-H, Shim E-J, Park J-H, Choi J-Y, Kim S G, Park E-C. (2010).
I wish to validate the above CNAT-C questionnaire in the Sri Lankan context (Sinhala version).
I would be much grateful to you if you could provide the English version of this scale (with the scoring system) and grant your kind permission to validate.
I am looking forward to your kind response.
Thank you
Best Regards
Eranthi
Ms. SMEB Weeratunga
RN, BScN, MPhil
Senior Lecturer
Department of Nursing
Faculty of Allied Health Sciences
University of Ruhuna
Mobile No. -

+94 77 2258519, +94 71 4402662
E-mail- eranthiweeratunga@yahoo.com, eranthiw@ahs.ruh.ac.lk

- CNAT for patient, caregiver.pdf
  76.5kB
